# Mitochondrial Toxicity Associated with Imatinib and Sorafenib in Isolated Rat Heart Fibers and the Cardiomyoblast H9c2 Cell Line

**DOI:** 10.3390/ijms23042282

**Published:** 2022-02-18

**Authors:** Jamal Bouitbir, Miljenko V. Panajatovic, Stephan Krähenbühl

**Affiliations:** 1 Division of Molecular and Systems Toxicology, Department of Pharmaceutical Sciences, University of Basel, Basel, Switzerland; 2 Division of Clinical Pharmacology & Toxicology, University Hospital, Basel, Switzerland; m.panajatovic@unibas.ch (M.V.P.); stephan.kraehenbuehl@usb.ch (S.K.)

**Keywords:** imatinib, sorafenib, cardiotoxicity, electron transfer system, reactive oxygen species, glutathione, apoptosis

## Abstract

Tyrosine kinase inhibitors (TKIs) are associated with cardiac toxicity, which may be caused by mitochondrial toxicity. The underlying mechanisms are currently unclear and require further investigation. In the present study, we aimed to investigate in more detail the role of the enzyme complexes of the electron transfer system (ETS), mitochondrial oxidative stress, and mechanisms of cell death in cardiac toxicity associated with imatinib and sorafenib. Cardiac myoblast H9c2 cells were exposed to imatinib and sorafenib (1 to 100 µM) for 24 h. Permeabilized rat cardiac fibers were treated with both drugs for 15 min. H9c2 cells exposed to sorafenib for 24 h showed a higher membrane toxicity and ATP depletion in the presence of galactose (favoring mitochondrial metabolism) compared to glucose (favoring glycolysis) but not when exposed to imatinib. Both TKIs resulted in a higher dissipation of the mitochondrial membrane potential in galactose compared to glucose media. Imatinib inhibited Complex I (CI)- and CIII- linked respiration under both conditions. Sorafenib impaired CI-, CII-, and CIII-linked respiration in H9c2 cells cultured with glucose, whereas it inhibited all ETS complexes with galactose. In permeabilized rat cardiac myofibers, acute exposure to imatinib and sorafenib decreased CI- and CIV-linked respiration in the presence of the drugs. Electron microscopy showed enlarged mitochondria with disorganized cristae. In addition, both TKIs caused mitochondrial superoxide accumulation and decreased the cellular GSH pool. Both TKIs induced caspase 3/7 activation, suggesting apoptosis as a mechanism of cell death. Imatinib and sorafenib impaired the function of cardiac mitochondria in isolated rat cardiac fibers and in H9c2 cells at plasma concentrations reached in humans. Both imatinib and sorafenib impaired the function of enzyme complexes of the ETS, which was associated with mitochondrial ROS accumulation and cell death by apoptosis.

## 1. Introduction

Tyrosine kinases are involved in cell proliferation, as well as tumorigenesis and progression, and have emerged as main targets for drug discovery. A tyrosine kinase inhibitor (TKI) is designed to inhibit the corresponding kinase from playing its role of catalyzing protein phosphorylation. Until the discovery of the TKIs, most treatments of non-resectable cancer involved cytotoxic chemotherapy and possibly radiation [1]. Compared to conventional cytotoxic chemotherapy, the introduction of TKIs on the market has improved patient survival and quality of life due to a higher efficacy and safety. Among TKIs, imatinib mesylate was the first low-molecular-weight anticancer drug approved by the US Food and Drug Administration (FDA) two decades ago. Imatinib (marketed as “Gleevec”) represented a revolution in the management of several cancers, including chronic myelogenous leukemia (CML), gastrointestinal stromal cell tumors (GISTs), and hypereosinophilic syndrome [2]. Imatinib inhibits the BCR-ABL fusion protein by competitively binding to the ATP-binding site of the protein [3]. In addition, this TKI inhibits the oncogenic platelet-derived growth factor receptor (PDGFR), stem cell factor (SCF), and the hepatocyte factor receptor (c-kit). In 2005, the multitarget TKI sorafenib (marketed as “Nexavar”) was approved for the treatment of patients with different cancers, including metastatic renal cell carcinoma, hepatocellular carcinoma, and imatinib-resistant gastrointestinal stromal cell tumors (GISTs) [4]. As a multitarget kinase inhibitor, sorafenib blocks tumor cell proliferation by inhibiting the activity of Raf-1, B-Raf, and kinases in the Ras/Raf/MEK/ERK-signaling pathway. Moreover, sorafenib can inhibit angiogenesis by targeting c-kit, FMS-like tyrosine kinase (FLT-3), vascular endothelial growth factor receptor VEGFR-2, VEGFR-4, PDGFR-β, and other tyrosine kinases [5].

Despite the targeted approach, imatinib and sorafenib frequently cause unwanted side effects, including fatigue, rash, myelosuppression, gastrointestinal toxicity, skeletal muscle toxicity, and hepatotoxicity [6]. Moreover, adverse effects of TKIs also include cardiotoxicity, which can be manifested by a multitude of cardiovascular complications. Adverse cardiovascular events observed with imatinib and sorafenib include hypertension, arrhythmias, QT prolongation, congestive heart failure (CHF), reduced left ventricular ejection fraction (LVEF), which are not well-predicted by standard preclinical analyses and are hence unanticipated [7,8,9,10,11,12,13,14]. The importance of these cardiovascular toxicities is not due to their frequency but rather due to their potentially large impact on patients.

To date, the molecular mechanisms of TKI-associated cardiotoxicity have yet to be fully understood. Multiple lines of evidence have suggested mitochondrial dysfunction as a mechanism for TKI-induced toxicity in general [15,16,17,18,19,20], as well as cardiotoxicity. In normal cardiomyocytes, sorafenib was reported to inhibit mitochondrial respiration and to decrease intracellular ATP [21]. Similarly, we recently showed that sunitinib is a mitochondrial toxicant in cardiomyoblast H9c2 cells and in mouse hearts [22]. Cardiomyocyte is a cell type with the highest content of mitochondria in the mammalian body, reflecting the high ATP turnover of the organ due to its permanent contractile activity. Accordingly, mitochondria may represent a key factor for cardiotoxicity associated with TKIs [21,23]. Drugs interfering with oxidative phosphorylation (OXPHOS) may deplete ATP stores and lead to myocardial dysfunction. Mitochondrial damage may be induced by impairing the respiratory electron transfer system (RETS) by depleting the mitochondrial membrane potential and/or by increasing oxidative stress. However, the underlying mechanism of imatinib- and sorafenib-induced cardiotoxicity merits further investigation. For instance, little is known regarding which enzyme complex(es) of the electron transfer system (ETS) is (are) inhibited by imatinib and sorafenib. A study of the role of mitochondrial oxidative stress pathways and apoptosis concerning imatinib- and sorafenib-induced cardiotoxicity is needed, as well as the identification of key signaling proteins and biomarkers, which could potentially provide possibilities for interventions in cardiotoxicity caused by imatinib and sorafenib.

Based on these considerations, we used permeabilized cardiac fibers from rats and the rat cardiomyoblast H9c2 cell line and to examine the short- and long-term effects of imatinib and sorafenib on mitochondrial respiratory capacities. We investigated short- and long-term exposure to imatinib and sorafenib since the mechanisms of toxicity may depend on duration of exposure. We then evaluated the effect of mitochondrial oxidative metabolism on the generation of mitochondrial oxidative stress and apoptosis as potential mechanisms of imatinib- and sorafenib-associated cardiotoxicity.

## 2. Results

### 2.1. Membrane Toxicity and Intracellular ATP Content in Imatinib- and Sorafenib-Treated H9c2 Cells

**Figure 1:**
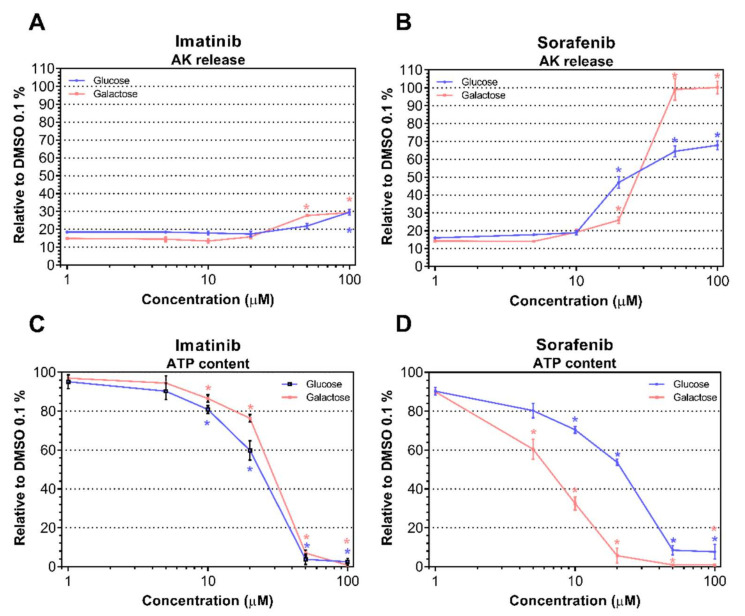
Membrane toxicity and intracellular ATP content in H9c2 cells exposed to imatinib and sorafenib. (**A**,**B**) Membrane toxicity was assessed by the release of adenylate kinase (AK) after drug exposure for 24 h under glucose and galactose media with imatinib (**A**) and sorafenib (**B**). Data are expressed as % AK release in the presence of 0.1% Triton X (set at 100%). (**C**,**D**) Intracellular ATP content after drug exposure for 24 h under glucose and galactose media with imatinib (**C**) and sorafenib (**D**). Data represent the percentage of ATP content in the presence of 0.1% DMSO (set at 100%). Data represent the mean ± SEM of at least four independent experiments. * *P* < 0.05 versus 0.1% DMSO control.

We first assessed plasma membrane integrity and intracellular ATP content in H9c2 cells exposed to imatinib and sorafenib at concentrations between 1 and 100 µM for 24 h using glucose and galactose media. Under the galactose condition, we were able to force the cells to produce ATP, mainly via oxidative phosphorylation, whereas under the glucose condition, cells were able to produce ATP via both oxidative phosphorylation and glycolysis [24,25]. In H9c2 cells exposed for 24 h, imatinib and sorafenib were membrane-toxic and diminished the cellular ATP pool in a concentration-dependent manner. Imatinib was membrane-toxic starting at 100 µM under glucose (*P* = 0.0002) and starting at 50 µM under galactose media (*P* < 0.0001; Figure 1A). Interestingly, this TKI depleted intracellular ATP content starting at 10 µM for both conditions (*P* = 0.012 for glucose condition and *P* = 0.0074 for galactose condition; Figure 1C). Sorafenib was membrane-toxic starting at 20 µM for both media (*P* < 0.0001 for both conditions; Figure 1B), and intracellular ATP stores decreased starting at 10 µM for glucose (*P* < 0.0001) and 5 µM for galactose (*P* < 0.0001; Figure 1D). Then, we calculated the corresponding IC_50_ values for each drug and for each condition indicated in Table 1. More pronounced mitochondrial toxicity can be expected in the presence of galactose as compared to glucose, and ATP depletion was observed at lower concentrations than membrane toxicity [24,25]. For imatinib, we found that membrane toxicity and ATP depletion started almost at the same concentrations under glucose and galactose media (Table 1). In comparison, sorafenib was more membrane-toxic in the presence of galactose than glucose. Moreover, a ratio IC_50_ ATPglu/IC_50_ ATPgal > 2 is regarded as an indicator of mitochondrial toxicity [26]. Accordingly, we assumed mitochondrial toxicity for sorafenib but not for imatinib. Nevertheless, we decided to investigate the mechanisms of toxicity of both TKIs.

### 2.2. TMRM Fluorescence Intensity as a Marker for Mitochondrial Membrane Potential in H9c2 Cells Exposed to Imatinib and Sorafenib

**Figure 2:**
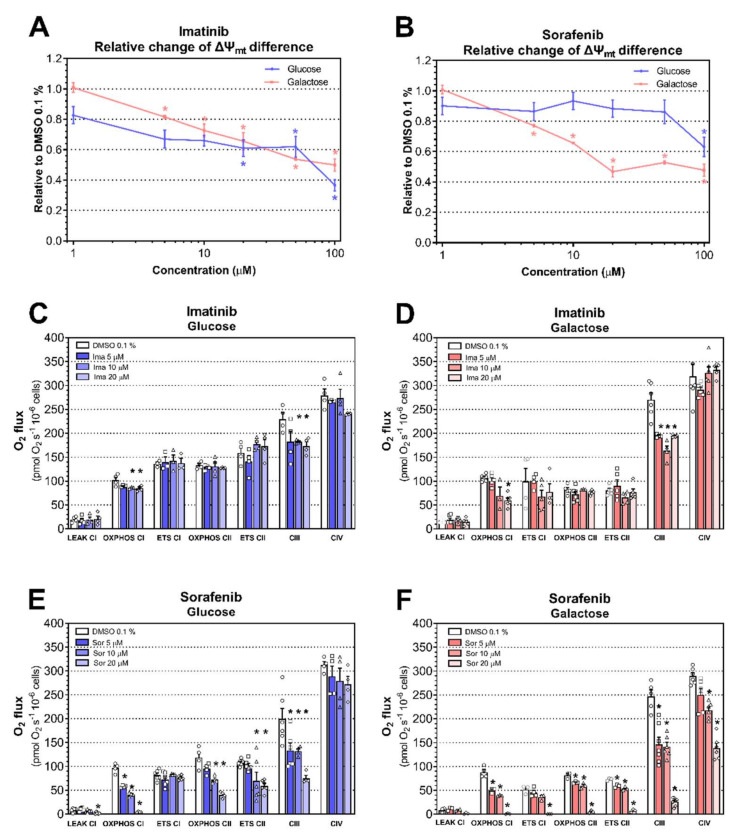
Effects of imatinib and sorafenib on TMRE fluorescence intensity for mitochondrial membrane potential and on mitochondrial respiratory capacities. (**A**,**B**) Relative change of mitochondrial membrane potential difference in H9c2 cells exposed to imatinib (**A**) and to sorafenib (**B**) under glucose and galactose media. (**C**,**D**) Respiratory capacities through complexes of the ETS in H9c2 cells exposed to imatinib for 24 h under glucose (**C**) and galactose (**D**) media. (**E**,**F**) Respiratory capacities through complexes of the ETS in H9c2 cells exposed to sorafenib for 24 h under glucose (**E**) and galactose (**F**) media. Data represent the mean ± SEM of at least four independent experiments. * *P* < 0.05 versus 0.1% DMSO control.

As a next step, we evaluated the effect of imatinib and sorafenib on TMRM fluorescence intensity as a marker for mitochondrial membrane potential in H9c2 cells [27]. Imatinib decreased TMRM fluorescence intensity starting at 20 µM under glucose conditions (*P* = 0.039), compared to 5 µM under galactose conditions (*P* = 0.031; Figure 2A). We observed a dissipation in TMRM fluorescence intensity starting at 100 µM for sorafenib under glucose (*P* = 0.032) and at 5 µM under galactose conditions (*P* = 0.0001; Figure 2B). These data confirmed the role of mitochondrial toxicity for sorafenib and suggested that mitochondrial damage could contribute to the toxicity of imatinib. However, we cannot exclude a direct effect of imatinib and sorafenib on the plasma membrane potential with this assay.

### 2.3. Mitochondrial Respiration in H9c2 Cells and in Rat Permeabilized Cardiac Fibers Exposed to Imatinib and Sorafenib

In order to support these data, we determined respiratory capacities through the complexes of the ETS in H9c2 cells using a high-resolution respirometry system. We measured the respiratory capacities in three mitochondrial-coupling states: LEAK state (native respiration in the absence of phosphorylation), OXPHOS state (fully active phosphorylation system), and ETS state (electron transfer uncoupled from the phosphorylation system). Under glucose and galactose media, imatinib elicited no impact on LEAK state (Figure 2C,D, respectively). Under the glucose condition, imatinib inhibited OXPHOS CI- and CIII-linked capacity starting at 10 µM (Figure 2C). Under the galactose condition, imatinib inhibited OXPHOS CI capacity starting at 20 µM and decreased CIII-linked respiration starting at 5 µM (Figure 2D). Interestingly, imatinib did not affect OXPHOS CII- and CIV-linked respiration under either media. Moreover, the maximal oxidative capacity (ETS CI and ETS CII capacity) was not affected by the exposure of imatinib to H9c2 cells under either media (Figure 2C,D). Under the glucose condition, sorafenib inhibited LEAK state at 20 µM and decreased OXPHOS CI-, OXPHOS CII-, and CIII-linked respiration starting at 5 µM (Figure 2E). Moreover, ETS CII capacity was lower in cells under glucose exposed to sorafenib starting at 10 µM (Figure 2E). Under the galactose condition, sorafenib impaired OXPHOS CI-, OXPHOS CII-, CIII-, and CIV-linked respiration in a concentration-dependent fashion starting at 5 µM, except for CIV, starting at 10 µM (Figure 2F). Furthermore, ETS CI capacity was decreased by sorafenib starting at 20 µM and 5 µM for ETS CII capacity (Figure 2F).

**Figure 3:**
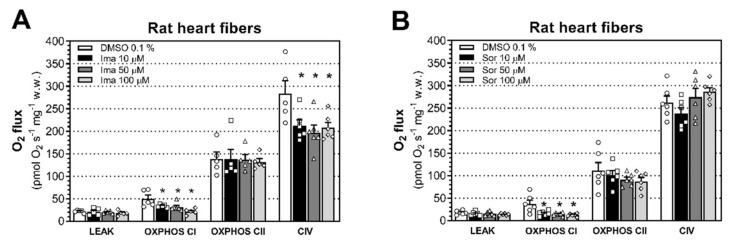
Effects of acute exposure of imatinib and sorafenib on mitochondrial respiratory capacities. We exposed permeabilized cardiac fibers from rats to imatinib (**A**) and sorafenib (**B**) for 15 min and then measured respiratory capacities through complexes of the ETS in the presence of the drugs or DMSO 0.1%. Data represent the mean ± SEM of at least five independent experiments. * *P* < 0.05 versus 0.1% DMSO control.

In order to confirm these results, we isolated cardiac fibers from wild-type rats, which we directly exposed to imatinib and sorafenib for 15 min in order to investigate the acute effect of these toxicants on mitochondrial respiration. Then, we measured respiratory capacities through the complexes of the ETC using an HRR system in the presence of the drugs in the chambers of an Oxygraph-2k. We found that this acute exposure starting at 10 μM for imatinib was associated with decreased OXPHOS CI-linked respiration (*P* = 0.029) and CIV-linked respiration (*P* = 0.032; Figure 3A). Short-term exposure of permeabilized cardiac fibers was also associated with decreased OXPHOS CI-linked substrate state starting at 10 μM for sorafenib (*P* = 0.0074; Figure 3B).

### 2.4. Oxidative Stress in H9c2 Cells Exposed to Imatinib and Sorafenib

**Figure 4:**
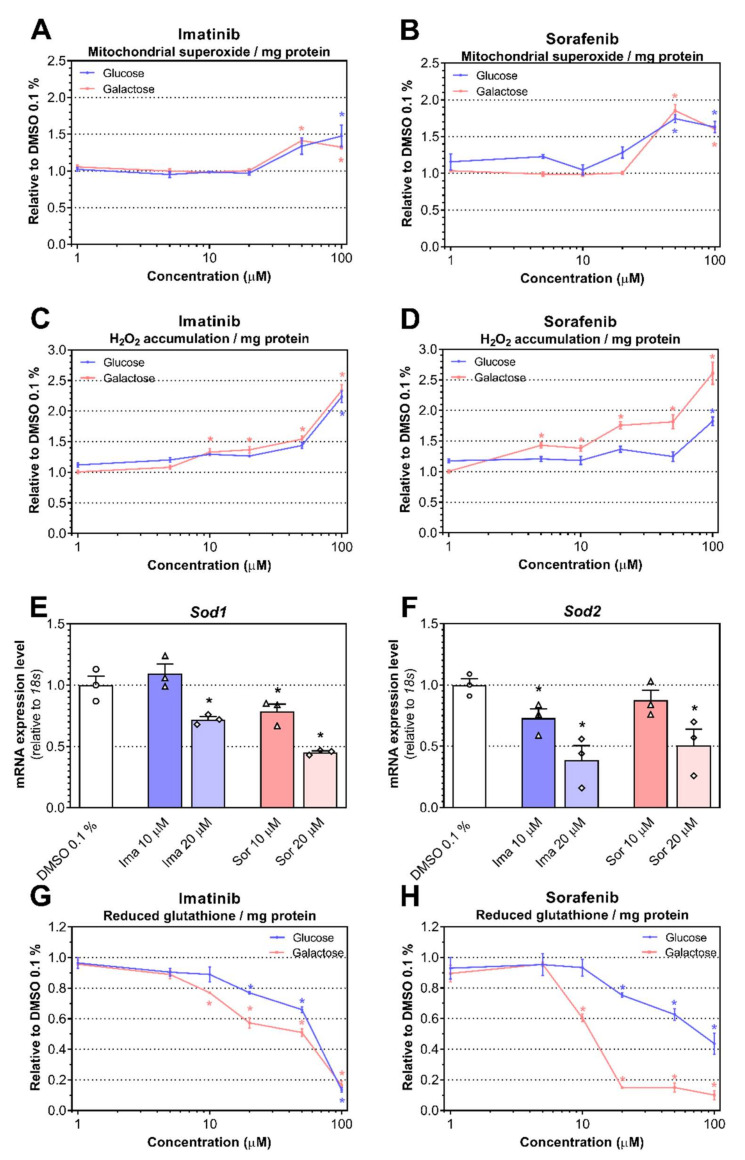
Effects of imatinib and sorafenib on redox status. (**A**,**B**) Mitochondrial superoxide content after 24 h exposure to imatinib (**A**) and sorafenib (**B**) under glucose and galactose media. (**C**,**D**) Cellular H_2_O_2_ content after 24 h exposure to imatinib (**C**) and sorafenib (**D**) under glucose and galactose media. (**E**,**F**) Relative mRNA expression of *Sod1* (**E**) and *Sod2* (**F**) of cells exposed to imatinib (Ima) and sorafenib (Sor) under glucose media. (**G**,**H**) GSH content after 24 h exposed to imatinib (**E**) and sorafenib (**F**) under glucose and galactose media. Data represent the mean ± SEM of at least four independent experiments. * *P* < 0.05 versus 0.1% DMSO control.

When the CI and CIII of the ETS are inhibited, they can increase superoxide production within mitochondria, which is then dismutated to hydrogen peroxide (H_2_O_2_) by superoxide dismutases (SOD) [28,29]. Accordingly, we measured the accumulation of superoxide anion in H9c2 cells grown either in glucose- or galactose-supplemented media and exposed to imatinib and sorafenib for 24 h. We found increased mitochondrial superoxide accumulation starting at 50 µM when H9c2 cells were exposed to imatinib cultured in galactose (*P* < 0.0001) but at 100 µM for imatinib cultured in glucose (*P* = 0.005; Figure 4A). Antimycin A (100 µM), an inhibitor of CIII of the ETS, was used as a positive control and increased superoxide accumulation under both conditions (3.9 ± 0.8 for glucose and 5.1 ± 1.4 for galactose; *P* < 0.0001 for both conditions). As a second marker of ROS generation, we measured cellular production of H_2_O_2_. The positive control (20 µM menadione) increased H_2_O_2_ production in both media (6.4 ± 1.9 for glucose and 6.9 ± 1.1 for galactose; *P* < 0.0001 for both conditions). In low glucose-supplemented cells, imatinib and sorafenib started to increase cellular production of H_2_O_2_ at 100 µM (*P* = 0.0019 and *P* < 0.0001, respectively; Figure 4C,D, respectively), whereas under galactose condition, H_2_O_2_ production increased at 10 μM for imatinib (*P* = 0.0064) and 5 μM for sorafenib (*P* = 0.033; Figure 4C,D, respectively). Under low glucose, mRNA expression of *Sod1*, located in the cytosol and in the intermembrane space of mitochondria, decreased in cells exposed to imatinib starting at 20 μM (*P* = 0.016), whereas mRNA expression of *Sod2*, located exclusively in the mitochondrial matrix, decreased starting at 10 μM (*P* = 0.040; Figure 4E,F, respectively). In H9c2 cells exposed to sorafenib, *Sod1* mRNA expression decreased starting at 10 μM (*P* = 0.049) whereas *Sod2* mRNA expression decreased at 20 μM (*P* = 0.025; Figure 4E and 4F, respectively). Another line of defense against ROS accumulation is represented by the glutathione antioxidant system, which can scavenge ROS [30,31]. Imatinib and sorafenib depleted GSH content starting at 20 μM in the presence of glucose (*P* = 0.011 and *P* = 0.012, respectively) and at 10 μM in the presence of galactose (*P* = 0.0009 and *P* = 0.002, respectively; Figure 4G,H, respectively). As a positive control, 100 µM buthionine sulfoximine (BSO) was used and decreased the GSH content by more than 90% in both media (*P* < 0.0001).

### 2.5. Mitochondrial Morphology and Content in H9c2 Cells Exposed to Imatinib and Sorafenib

**Figure 5:**
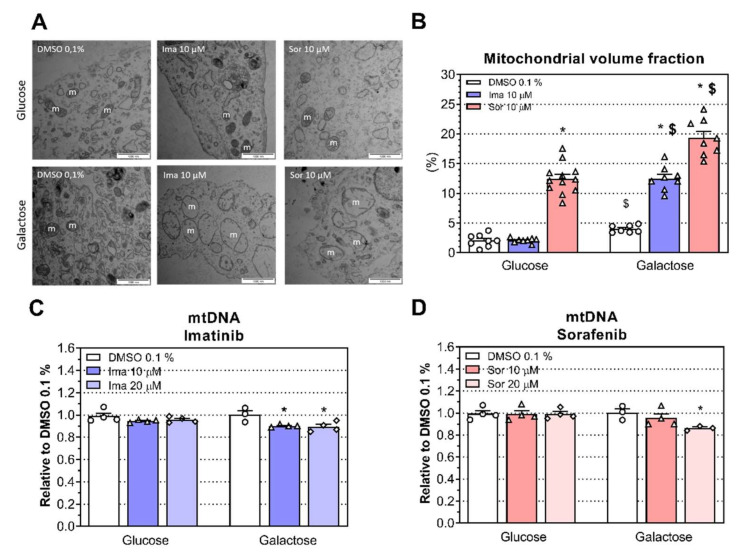
Mitochondrial morphology and mtDNA content of H9c2 cells exposed to imatinib (Ima) and sorafenib (Sor) for 24 h under glucose and galactose media. (**A**) Transmission electron microscopy images of H9c2 cells exposed to 10 μM imatinib and 10 μM sorafenib under glucose and galactose conditions (m: mitochondrion; scale bar = 1000 nm). (**B**) Mitochondrial volume fraction in H9c2 cells. (**C**) Mitochondrial DNA content in H9c2 cells exposed to imatinib at 10 μM and 20 μM for 24 h under glucose and galactose media. (**D**) Mitochondrial DNA content in H9c2 cells exposed to sorafenib at 10 μM and 20 μM for 24 h under glucose and galactose media. Data represent the mean ± SEM of at least three independent experiments. * *P* < 0.05 treatment groups versus 0.1% DMSO control; $ *P* < 0.05 galactose versus glucose of the same treatment group.

Transmission electron microscopy (TEM) was used to visualize changes in mitochondrial morphology in H9c2 cells exposed to 10 μM imatinib and 10 μM sorafenib for 24 h. Cells treated with 10 μM imatinib or 10 μM sorafenib under both conditions presented mitochondrial alterations such as an increase in mitochondrial size and disorganization and partial destruction of mitochondrial cristae (Figure 5A). Accordingly, H9c2 cells treated with 10 μM imatinib had a higher mitochondrial volume fraction than control incubations under galactose (*P* < 0.0001) but not under glucose conditions (Figure 5B). In comparison, sorafenib increased the mitochondrial volume fraction at 10 μM for both conditions compared to the respective controls (*P* < 0.0001 for both media; Figure 5B). However, the mitochondrial volume fraction was higher under galactose compared to low-glucose conditions when cells were exposed to 10 μM imatinib or sorafenib (*P* < 0.0001). In addition, we measured mtDNA content as an indirect marker of mitochondrial content. Imatinib and sorafenib decreased mtDNA content at 10 μM and 20 μM, respectively, under galactose (*P* = 0.024 and *P* = 0.047, respectively) but not under glucose conditions (Figure 5C,D, respectively).

### 2.6. Cell Death in H9c2 Cells Exposed to Imatinib and Sorafenib

**Figure 6:**
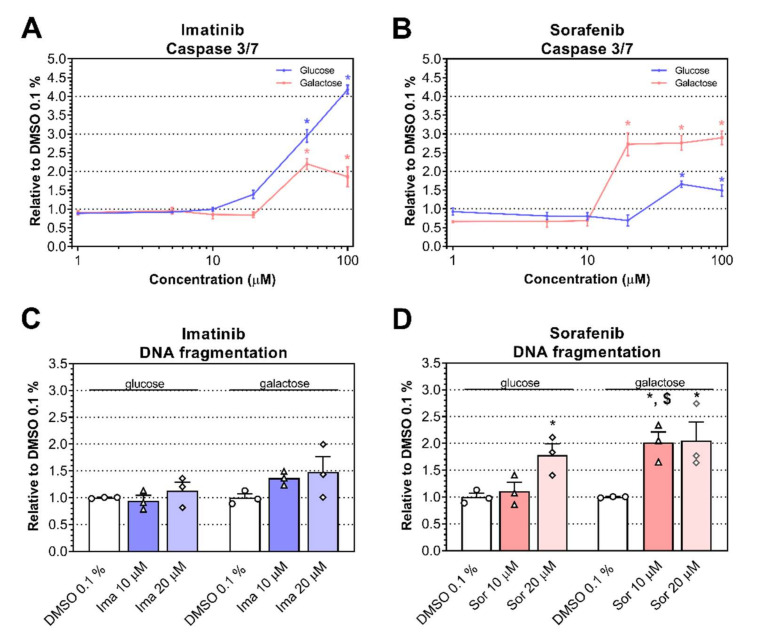
Effects of imatinib (Ima) and sorafenib (Sor) on markers of apoptosis in H9c2 cells under glucose and galactose media. (**A**,**B**) Caspase 3/7 activity after exposure to imatinib (**A**) and sorafenib (**B**) for 24 h under glucose and galactose media. (**C**,**D**) DNA fragmentation observed in cardiomyoblast H9c2 cells after exposure to imatinib (**C**) and sorafenib (**D**) for 24 h under glucose and galactose media. Data represent the mean ± SEM of at least three independent experiments. * *P* < 0.05 versus 0.1% DMSO control; $ *P* < 0.05 galactose versus glucose of the same treatment group.

When oxidative stress overwhelms antioxidative defenses, it induces damage to proteins, lipids, and DNA, which can then provoke apoptosis. To find out whether apoptosis is associated with imatinib and sorafenib, we measured the activity of caspase 3/7. Imatinib increased the activity of caspase 3/7 starting at 50 μM for both conditions (*P* = 0.039 for glucose and *P* = 0.0025 for galactose). Sorafenib increased the activity of caspase 3/7 starting at 20 μM under galactose (*P* = 0.0009) but only at 50 μM under glucose (*P* = 0.014), suggesting increased apoptosis (Figure 6A,B, respectively). As a second marker of cell death, we evaluated DNA fragmentation in H9c2 cells. DNA fragmentation was not detectable up to 20 μM for imatinib under either condition (Figure 6C). Under the glucose condition, DNA fragmentation increased for sorafenib starting at 20 μM (*P* = 0.021), whereas under the galactose condition, DNA fragmentation started to increase at 10 μM (*P* = 0.037; Figure 6D). Moreover, DNA fragmentation was higher under galactose compared to low-glucose conditions when cells were exposed to 10 μM of sorafenib (*P* = 0.024).

## 3. Discussion

Imatinib and sorafenib are associated with cardiac toxicity, such as arrhythmias, congestive heart failure, QT prolongation, and heart ischemia. The underlying mechanisms of these cardiac adverse effects have yet to be fully elucidated. In the current study, we provided evidence that acute exposure of isolated rat heart fibers to imatinib and sorafenib impaired OXPHOS CI-linked respiration. In addition, short-term exposure to imatinib also inhibited CIV-linked respiration. Furthermore, H9c2 cells cultured in glucose or galactose media exposed to imatinib or sorafenib for 24 h showed a drop in intracellular ATP content and dissipation of mitochondrial membrane potential. Detailed analysis of the RETS indicated that imatinib inhibited CI- and CIII-linked respiration, whereas sorafenib impaired CI-, CII-, and CIII-linked respiration. Both drugs caused mitochondrial ROS accumulation, which was associated with depletion of glutathione and apoptosis of H9c2 cells.

In our study, we used glucose and galactose media. Normal mammalian cells obtain ATP from both glycolysis and OXPHOS. In comparison, cancer cells mainly obtain ATP from glycolysis rather than OXPHOS in the presence of glucose. Despite the presence of functional mitochondria, the Crabtree effect (favoring ATP production by glycolysis) supports the survival of cancer cells in metabolic stress conditions [32]. In cancer cells, the replacement of glucose with galactose shifts ATP generation towards OXPHOS because glycolytic degradation of galactose via glucose yields no net ATP. Cells grown in the presence of galactose instead of glucose were shown to increase expression of OXPHOS constituents and to become sensitized to mitochondrial toxicants [24]. Sensitization of H9c2 cells to mitochondrial toxicants was the main reason to replace glucose with galactose in the current investigation.

Imatinib reduced the ATP content of H9c2 cells starting at 10 µM under both culture conditions and impaired membrane integrity starting at 100 and 50 µM under glucose and galactose conditions, respectively. The concentration difference between the start of cellular ATP depletion and membrane toxicity suggested mitochondrial toxicity [25]. However, the IC_50_ for ATP depletion between glucose and galactose media was in the same range. Our findings are comparable to those of Will et al., who reported ATP depletion in H9c2 cells exposed to imatinib for 24 h starting at 30 µM [21], which is slightly higher than in the current study. In comparison, in neonatal rat ventricular myocytes, ATP depletion started at 5 µM imatinib [8], suggesting that the extent of mitochondrial toxicity of imatinib depends on the cell type used and possibly on the culture conditions. In previous studies, changes in the morphology of mitochondria and endoplasmic reticular (ER) membranes were observed in biopsies of humans and in mice treated with imatinib [8]. In these studies, the mechanism of mitochondrial toxicity of imatinib was considered to be mainly a consequence of ER stress, which impaired the import of proteins into mitochondria [4,8]. Ultrastructural changes in mitochondria were also observed in hearts of imatinib-treated patients [8] similar to those observed in the current investigation. Studies with permeabilized heart muscle fibers showed a decrease in respiratory capacity after 15 min of exposure to imatinib. This finding suggests a direct toxicity on CI and CIV, which appears to be an early consequence of the described defect in protein import due to ER stress. When inhibited, CI and CIII are the main source of superoxide anions within mitochondria [28]. For this reason, imatinib increased mitochondrial ROS production and accumulation, which was responsible for apoptosis of H9c2 cells.

Typical plasma concentrations of imatinib in humans are in the range of 3 to 5 μM [33], whereas we observed impairment of respiratory capacities starting at 10 μM. Imatinib is heavily metabolized by CYP3A4; considering the high variability of CYP3A4 in the population and the existence of CYP3A4 inhibitors, it appears to be possible that some patients can reach cardiac imatinib concentrations in the toxic range.

Sorafenib increased membrane toxicity starting at 20 µM under both culture conditions and reduced ATP content starting at 10 and 5 µM under glucose and galactose conditions, respectively. Since the ratio IC_50_ ATPglu/IC_50_ ATPgal was higher than 2.0, we expected mitochondrial toxicity for sorafenib. Accordingly, we found that the inhibition of CI-, CII-, and CIII-linked respiration started at 5 μM, whereas mitochondrial ROS accumulation and activation of caspase 3/7 started at 50 µM. Our data confirm recently published results showing increased mitochondrial ROS in isolated rat ventricular myocytes [34]. However, a previous study of human cardiomyocytes demonstrated an increase in ROS at the cellular but not at the mitochondrial level [35]. This discrepancy can be explained by the different cell models used and/or the different methods used for determination of ROS accumulation. In our study, we measured mitochondrial and cellular ROS accumulation, as well as markers of the antioxidant system, such as glutathione and superoxide dismutase activities, in order to obtain a more complete picture of the redox state of H9c2 cells exposed to TKIs.

Sorafenib has been described to reduce systolic function in mice [36,37], to impair contractility of rat heart tissue [38], and to alter mitochondrial morphology in rats [23]. Furthermore, reversible cardiac toxicity of sorafenib has been described in human patients [9,14]. Studies in human patients [39] and in rats [23] suggest that sorafenib is a toxicant for heart mitochondria. The current study confirmed these findings and showed that sorafenib inhibits CI-, CII-, and CIII-linked respiration in H9c2 cells, which was responsible for the accumulation of mitochondrial ROS at relevant concentrations. In comparison, in a previous investigation sorafenib inhibited only CIII-linked respiration in isolated cardiomyocytes and impaired the activities of both CII- and CIII-linked respiration in HeLa cells [40]. One explanation for the discrepancy in results in the current study is the method used for the determination of mitochondrial respiration. In the current study, we used high-resolution respirometry with an Oroboros 2k-Oxygraph system, which is considered to provide the best resolution and sensitivity in biological samples [41,42]. We also have to take account that these studies used different cell lines and that the duration of exposure with TKIs was different. 

Similarly to imatinib, sorafenib was also acutely toxic to cardiomyocytes by directly inhibiting CI-linked respiration. Acute toxicity was concentration-dependent and started at 10 μM in isolated cardiac myofibers. Typical plasma concentrations in patients for sorafenib were approximately 5 μM [43,44], which is close to the concentration where we started to see impairment of mitochondrial respiratory capacity.

Based on the results of the current study, we propose a two-step mechanism regarding mitochondrial toxicity of imatinib and sorafenib. Acute exposure of myofibers to these drugs mainly impairs the function of complex I of the electron transfer system, which is associated with increased production of ROS and, depending on the strength of the insult, a drop in ATP production and a decrease in mitochondrial membrane potential [28]. The mechanism of the acute impairment of enzyme complexes of the electron transfer system by these amphiphilic compounds is not exactly known but may be related to accumulation of these drugs in the inner mitochondrial membrane. This may disturb the function of proteins located in this membrane, such as enzyme complexes of the electron transfer system and/or transporters of the substrates used for mitochondrial metabolism. In a second step, when ROS production exceeds the mitochondrial antioxidative defense mechanisms, ROS start to accumulate in mitochondria, leading to a decrease in mitochondrial and cellular glutathione stores and impaired function of mitochondrial proteins and mitochondrial DNA. As shown in the current investigation, in contrast to acute toxicity, this type of toxicity cannot be reversed by removal of the toxic compounds.

## 4. Materials and Methods

### 4.1. Chemicals

Imatinib mesylate and sorafenib were purchased from Sequoia Research Products (Pangbourne, UK). Stock solutions were prepared in dimethylsulfoxide (DMSO) and stored at −20 °C. All other chemicals were supplied by Sigma-Aldrich (Buchs, Switzerland), except where indicated.

### 4.2. Cell Culture

H9c2 cells were provided by Dr. Pfister (University Hospital, Basel, Switzerland). H9c2 cells were kept under low-glucose and galactose media. In the low-glucose condition, H9c2 cells were cultured in Dulbecco’s modified Eagle medium (DMEM) containing 5.55 mM (1 g/L) glucose supplemented with 10% (*v*/*v*) heat-inactivated fetal bovine serum, 1 mM sodium pyruvate, 4 mM GlutaMax, 5 mM HEPES buffer, 100 U/mL penicillin, and 100 µg/mL streptomycin (Invitrogen, Basel, Switzerland). In the galactose condition, H9c2 cells were cultured in Dulbecco’s modified Eagle medium (DMEM, containing no glucose) from Invitrogen (Basel, Switzerland) supplemented with 10% (*v*/*v*) heat-inactivated fetal bovine serum, 10 mM galactose, 5 mM HEPES buffer, 4 mM GlutaMax, 1 mM sodium pyruvate, 100 U/mL penicillin, and 100 µg/mL streptomycin. All cells were kept at 37 °C in a humidified 5% CO_2_ cell culture incubator and passaged using trypsin. We determined cell number with a Neubauer hemacytometer and cell viability with a trypan blue exclusion method. Cells used for the incubations with the toxicants were >95% viable. In control incubations, viability remained >95% after the final incubation for 24 h. 

### 4.3. Membrane Toxicity in H9c2 Cells

A Toxilight assay from Lonza (Basel, Switzerland) was used to assess membrane toxicity according to the manufacturer’s protocol. The release of adenylate kinase (AK) was measured in the medium, reflecting the integrity of the plasma membrane. H9c2 cells were treated for 24 h with 1 to 100 μM imatinib and sorafenib in the presence of glucose or galactose media. After this incubation time, we removed 20 μL of supernatant from each well and then transferred the supernatant into a new opaque 96-well plate. An amount of 100 μL of assay buffer was then added to each well. After 5 min incubation, luminescence was measured using a Tecan M200 Pro Infinity plate reader (Männedorf, Switzerland). A volume of 0.1% Triton X was used as a positive control. All data were normalized to positive control incubations containing 0.1% Triton X (set at 100% cell lysis).

### 4.4. Intracellular ATP Content in H9c2 Cells

We used a CellTiter-Glo Luminescent cell viability assay from Promega (Wallisellen, Switzerland) for determination of intracellular ATP in accordance with the manufacturer’s instructions. Cells were seeded at 15,000 cells/well in a 96-well plate 24 h prior drug treatment. H9c2 cells were treated with 1 to 100 μM imatinib and sorafenib for 24 h in the presence of glucose and galactose media. We added 50 μL of assay buffer to each 96-well plate containing H9c2 cells in 50 μL culture medium. After 15 min incubation, we measured the luminescence using a Tecan M200 Pro Infinity plate reader (Männedorf, Switzerland). Results were normalized to the 0.1% DMSO control condition.

### 4.5. Calculations of IC_50_ for Membrane Toxicity and ATP Content in H9c2 Cells

We calculated the IC_50_ for membrane toxicity (IC_50_-MT; drug concentration that causes a 50% loss in membrane integrity compared to the positive control Triton-X) and cellular decrease in ATP content (IC_50_-ATP; drug concentration that causes a 50% decrease in ATP content compared to 0.1% DMSO control) according to a previous publication [26] based on the data shown in Figure 1. Ratios of IC_50_-ATPglu/IC_50_-ATPgal or IC_50_-MTglu/IC_50_-MTgal > 2 were considered indicators of mitochondrial toxicity.

### 4.6. TMRM Fluorescence Intensity for Mitochondrial Membrane Potential in H9c2 Cells

We determined the fluorescence intensity of tetramethylrhodamine methyl ester (TMRM, Invitrogen, Basel, Switzerland) as a marker for mitochondrial membrane potential in H9c2 cells as described previously [27]. H9c2 cells were treated for 24 h with 1 to 100 μM imatinib and sorafenib under glucose and galactose media. After treatment with the drugs, cells were trypsinized. The cell pellet was then washed and resuspended in Dulbecco’s phosphate buffered saline (DPBS). TMRM was dissolved in DMSO and added to the resuspended cells at a final concentration of 50 nM. After 15 min incubation in the dark under agitation, cells were centrifuged and resuspended in DPBS for analysis with flow cytometry using a FACS Calibur cytometer (BD Bioscience, Allschwil, Switzerland). As an uncoupler of mitochondrial oxidative phosphorylation, carbonyl cyanide-4-(trifluoromethoxy)phenylhydrazone (FCCP, 9 μM) was used as a positive control and was exposed to H9c2 cells for 30 min. Data were analyzed using CellQuest Pro 6.0 software (BD Bioscience, Allschwil, Switzerland). Results were normalized to the 0.1% DMSO control condition.

### 4.7. High-Resolution Respirometry in H9c2 Cells

H9c2 cells were exposed to 5, 10, and 20 µM imatinib and sorafenib for 24 h in glucose and galactose media. After exposure to the toxicants, cells were collected, centrifuged, and washed before incubation in MiR05 buffer. Mitochondrial respiration measurements were performed using an Oxygraph-2k (O2k) high-resolution respirometer (HRR) equipped with DatLab software (Oroboros Instruments, Innsbruck, Austria). Temperature was maintained at 37 ± 0.001 °C under constant stirring at 750 rpm. All experiments were performed with oxygen concentration in the range of 210–50 µM O_2_. If necessary, reoxygenation was performed by partially raising the chamber stopper for a brief air equilibration. Experiments were realized in MiR05 buffer containing 0.5 mM EGTA, 3 mM magnesium chloride, 20 mM taurine, 10 mM potassium dihydrogen phosphate, 20 mM HEPES, 110 mM sucrose, 1 g/L fatty-acid-free bovine serum albumin, and 60 mM lactobionic acid, with a pH of 7.4, as described in [45]. For permeabilization of the cells, we first determined the optimal concentration of digitonin by inducing the maximal permeabilization of the plasma membrane without affecting the outer or inner mitochondrial membrane, as described in [45]. After routine respiration was established in the presence of cells, we added digitonin (9 µg/million cells) for permeabilization of the plasma membrane. In a first protocol, we measured LEAK state in the presence of the NADH-linked substrates glutamate (10 mM) and malate (2 mM), which transfer electrons along CI into the Q-pool. Then, we measured the corresponding OXPHOS CI capacity by adding ADP (2 mM). Oligomycin (2.5 µM) was added for the inhibition of the ATP synthase, and then the uncoupler carbonyl cyanide p-trifluro-methoxyphenyl hydrazine (FCCP, 1 µM) enabled the determination of CI-linked ETS capacity (ETS CI). Inhibition of CI by rotenone (0.5 µM) revealed the residual oxygen consumption (ROX) and was used for correction for the chemical background. Then, CIII-linked respiration was measured by adding duroquinol (500 µM). In the second protocol, rotenone (0.5 µM) and succinate (10 mM) were added to fuel electrons to CII. ADP (2 mM) was added for determination of OXPHOS CII capacity. Oligomycin (2.5 µM) was then added for inhibition of the ATP synthase, and then FCCP (1 µM) enabled the determination of CII-linked ETS capacity (ETS CII). CIII was inhibited by antimycin A (2.5 μM). Complex IV-linked respiration was measured by first adding ascorbate (2 mM) and then N,N,N’,N’-Tetramethyl-1,4-phenylenediamine (TMPD; 0.5 mM). Chemical background oxygen consumption was measured after inhibition of CIV with potassium chloride (KCN; 1 mM) and was used for correction of the chemical background. All oxygen fluxes were corrected for instrumental background oxygen flux. Respiration rates are expressed as pmol O_2_ s^−1^ million cells^−1^.

### 4.8. Rats

The experiments were performed in accordance with the National Institute of Health *Guide for the Care and Use of Laboratory Animals* (NIH Publication No. 8023, revised 1978) and were approved on January 1, 2013 by the Cantonal Veterinary Office of Basel-Stadt, Switzerland (License number 1745). Male Sprague–Dawley rats (*n* = 6, 250–300 g) were obtained from Janvier Labs (Saint-Berthevin, France) and housed in a standard facility with 12 h light–dark cycles and controlled temperature (21–22 °C). The rats were fed a standard pellet chow and water ad libitum. The rats did not receive any treatment. The rats were anaesthetized with an intraperitoneal application of ketamine (160 mg kg^−1^) and xylazine (20 mg kg^−1^). Removal of the beating heart was performed immediately. The apex was then removed, and the left ventricle was dissected for preparation of permeabilized myofibers. The left ventricle was immediately conserved in ice-cold BIOPS buffer containing 10 mM Ca-EGTA buffer, 0.1 μM free calcium, 5.77 mM ATP, 6.56 mM MgCl_2_, 20 mM taurine, 15 mM phosphocreatine, 0.5 mM dithiothreitol, and 50 mM K-MES, pH 7.1 until analysis.

### 4.9. High-Resolution Respirometry in Rat Cardiac Permeabilized Fibers

Mitochondrial oxygen consumption was studied in saponin-skinned fibers as described in [46]. Briefly, cardiac myofibers were separated under a binocular microscope in BIOPS buffer at 4 °C. After dissection, cardiac fibers were transferred and incubated at 4 °C for 30 min into BIOPS buffer containing 50 μg mL^−1^ saponin under constant shaking. Cardiac permeabilized fibers were then washed in BIOPS buffer for 10 min under intense shaking to completely remove saponin. All oxygen measurements were performed at 37 °C with an Oxygraph-2k apparatus equipped with DatLab software (Oroboros Instruments, Innsbruck, Austria) [45]. Immediately after measurement of wet mass (2–3 mg), the sample was transferred into 2.0 mL of MiR05 buffer, and we injected oxygen into the chambers to a level close to 400 μM, as described in [45]. We acutely exposed fibers to imatinib and sorafenib at 10, 50, and 100 μM for 15 min in the chambers. Then, the rate of basal respiration was measured with NADH-linked substrates ((glutamate (10 mM) + malate (2 mM)) before OXPHOS capacity was induced with ADP (2 mM). Under this condition, we measured CI-linked OXPHOS (OXPHOS CI). After inhibition of CI with rotenone (0.5 μM), respiration was restored with the addition of the CII substrate succinate (10 mM). Under these conditions, CII-linked OXPHOS (OXPHOS CII) was measured. Then, we inhibited CIII with antimycin A (2.5 μM), followed by the addition of artificial substrates for CIV: first ascorbate (2 mM) and then TMPD (0.5 mM). To verify the integrity of the outer mitochondrial membrane, we added cytochrome c (10 μM). The chemical background resulting from autoxidation of ascorbate, TMPD, and cytochrome c was determined in the absence of heart fibers in the chambers and was used for background correction [47]. Each experiment was performed in duplicate in the presence of the drugs. All oxygen fluxes were corrected for instrumental background oxygen flux. Respiration rates were expressed in pmol O_2_ s^−1^ mg^−1^ wet weight.

### 4.10. Mitochondrial Superoxide in H9c2 Cells

Mitochondrial superoxide was assessed using MitoSOX Red (Invitrogen, Basel, Switzerland) according to the manufacturer’s manual. H9c2 cells were seeded into black costar 96-well plates (20,000 cells/well). H9c2 cells were treated with 1 to 100 μM imatinib and sorafenib for 24 h in the presence of glucose or galactose media. The incubation with 100 µM antimycin A for 30 min was used as a positive control. After 24 h of exposure, cell culture medium was removed, and 2.5 µM MitoSOX dissolved in 100 µL Dulbecco’s phosphate-buffered saline (D-PBS) was added. After incubation for 10 min at 37 °C in the dark, fluorescence was measured (excitation, 510 nm; emission, 580 nm) using a Tecan M200 Pro Infinity plate reader (Männedorf, Switzerland). We normalized the results to the protein content using a Pierce Bicinchoninic acid protein assay kit (Thermo Fischer Scientific, Darmstadt, Germany) according to the manufacturer’s instructions. Results were normalized to the 0.1% DMSO control condition. 

### 4.11. Cellular Accumulation of H_2_O_2_ in H9c2 Cells

Production of reactive oxygen species (ROS) was assessed using a ROS-Glo assay (Promega, Wallisellen, Switzerland). H9c2 cells were treated with 1 to 100 μM imatinib and sorafenib for 24 h in the presence of glucose or galactose media. Menadione (20 µM) was used as a positive control. The assay was performed according to manufacturer’s manual, and luminescence was measured using a Tecan M200 Pro Infinity plate reader (Männedorf, Switzerland). Results were normalized to the 0.1% DMSO control condition. 

### 4.12. Cellular Reduced Glutathione (GSH) in H9c2 Cells

We used a luminescent GSH-Glo glutathione assay (Promega, Wallisellen, Switzerland) to measure the reduced form of glutathione (GSH). H9c2 cells were treated with 1 to 100 μM imatinib and sorafenib for 24 h under glucose and galactose media. We used 100 µM buthionine sulfoximine (BSO) as a positive control. The assay was performed according to the manufacturer’s manual. After 15 min incubation in the dark, luminescence was measured using a Tecan M200 Pro Infinity plate reader (Männedorf, Switzerland). Results were normalized to the 0.1% DMSO control condition.

### 4.13. Quantitative RT-PCR in H9c2 Cells

H9c2 cells were treated with 10 and 20 μM imatinib and sorafenib for 24 h in the presence of glucose medium. Total RNA was extracted from cells and purified using a Qiagen RNeasy mini extraction kit (Qiagen, Hombrechtikon, Switzerland). The quantity and purity of RNA were measured with a NanoDrop 2000 spectrophotometer (Thermo Scientific, Wohlen, Switzerland). cDNA was synthesized from 1 µg RNA using the Qiagen omniscript system. Real-time PCR measurement of individual cDNA was performed in triplicate using SYBR green dye (Roche Diagnostics, Rotkreuz, Basel) containing 10 μM of each primer (sense and antisense). The sequences of primer sets used are listed in Table 2. Relative mRNA expression levels were calculated using the ∆∆CT method with *18s* gene as internal control, as previously described [48].

### 4.14. Mitochondrial DNA Content in H9c2 Cells

H9c2 cells were treated with 10 and 20 μM imatinib and sorafenib for 24 h in the presence of glucose or galactose media. We then determined the ratio of DNA content of the mitochondrial gene ND1 and the nuclear gene 36B4 as a measure of the mitochondrial DNA content (see Table 2 for primers) using quantitative RT-PCR as described in [49], with some modifications. Total DNA was extracted using a DNeasy Blood and Tissue Kit (Qiagen, Hombrechtikon, Switzerland) following the manufacturer’s instructions. The concentration of extracted DNA was measured spectrophotometrically at 260 nm with a NanoDrop 2000 spectrophotometer (Thermo Scientific, Wohlen, Switzerland). Then, DNA was diluted in RNase-free water to a final concentration of 10 ng/μL. The expression of mitochondrial and nuclear genes was evaluated using SYBR green real-time PCR (Roche Diagnostics, Rotkreuz, Basel) performed on an ABI PRISM 7700 sequence detector (PE Biosystems, Rotkreuz, Switzerland). Quantification was performed using the comparative-threshold cycle method [49].

### 4.15. Transmission Electron Microscopy in H9c2 Cells

H9c2 cells were treated with 10 μM imatinib and sorafenib for 24 h in the presence of glucose or galactose media. After removal of the medium, cells were fixed with 3% Karnofski paraformaldehyde and 0.5% glutaraldehyde in 10 mM PBS with a pH of 7.4 for 1 h. After washing with PBS, cells were post-fixed with 1% reduced osmium tetroxide for 40 min and then treated with 1% osmium tetroxide for 40 min. After serial dehydration in ethanol, cells were embedded in epoxy resin. We obtained thin sections (60 nm) with a microtome Ultracut E from Leica (Biosystems Switzerland AG, Muttenz, Switzerland). Sections were first stained with 6% uranyl acetate for 60 min, followed by staining with lead acetate for 2 min. For analysis of the samples, we used a Moragagni electron microscope from FEI (Hillsboro, OR, USA) at 80 kV. 

We performed a blind analysis of randomly numbered electron micrographs using ImageJ software (version 1.53, Madison, WI, USA). We conducted at least three independent experiments, each performed in triplicate. For each condition, we analyzed 30–40 cells. We first quantified the total area for each cell and then measured the total mitochondrial area per cell. To calculate the mitochondrial volume fraction (reported in % of the cell volume), the total mitochondrial surface was divided by the area of each cell and multiplied by 100, as described in [50].

### 4.16. Caspase 3/7 Activity in H9c2 Cells

Cells were seeded at 15,000 cells/well in a 96-well plate 24 h prior drug treatment. H9c2 cells were treated with 1 to 100 μM imatinib and sorafenib for 24 h under glucose and galactose media. We used a luminescent Caspase-Glo 3/7 assay for determination of caspase 3/7 activity (Promega, Wallisellen, Switzerland) according to the manufacturer’s instructions. Luminescence was measured using a Tecan M200 Pro Infinity plate reader (Männedorf, Switzerland). Results were normalized to the 0.1% DMSO control condition.

### 4.17. Quantitative DNA Fragmentation in H9c2 Cells

H9c2 cells were treated with 10 and 20 μM imatinib and sorafenib for 24 h under glucose and galactose media. After exposure to imatinib and sorafenib, we removed the media, and cells were washed twice with DPBS. Cells were then lysed with RIPA buffer (50 nM, Tris-HCl, pH 7.4, 150 mM NaCl, 1% Triton X-100, 0.5% sodium deoxycholate, 0.1 % sodium dodecyl sulfate, and 1 mM EDTA). After cell lysis, an aliquot of the supernatant was placed in streptavidin-coated wells and incubated with anti-histone biotin antibody and anti-DNA peroxidase-conjugated antibody for 2 h at room temperature (Sigma-Aldrich, Buchs, Switzerland). Afterwards, the sample was removed, and the wells were washed three times with incubation buffer. After the final wash, we added 100 μL of the substrate 2,2′-azino-di(3-ethylbenzthiazolin-sulfonate) in each well for 20 min at room temperature. Absorbance was measured at 405 nm using a plate reader. Results were expressed as absorbance at 405 nm per minute per mg protein.

### 4.18. Statistical Analysis

Data are given as the mean ± SEM of at least three independent experiments. Statistical analyses were performed using GraphPad Prism 8 (GraphPad Software, La Jolla, CA, USA). For comparison of more than two groups, one-way ANOVA was used, followed by comparison between treatments containing test compounds and the control group using Dunnett’s post-test procedure. *P*-values < 0.05 (*) were considered significant.

## 5. Conclusions

In conclusion, imatinib and sorafenib impair the function of mitochondria in isolated rat cardiac myofibers and in H9c2 cells starting close to plasma concentrations detected in humans treated with these compounds. Acute damage to enzyme complexes of the mitochondrial electron transfer system, which may be due to accumulation of the toxicants in the inner mitochondrial membrane, causes mitochondrial ROS accumulation and secondary damage to mitochondrial proteins and function, eventually leading to apoptosis in H9c2 cells. The development and application of ROS scavengers specifically targeted to mitochondria could represent a strategy to prevent this type of toxicity.

## Figures and Tables

**Table 1:** Quantification of membrane toxicity and ATP depletion by imatinib and sorafenib in H9c2 cells.

	IC_50_ MT (μM)		IC_50_ ATP (μM)		IC_50_MTglu/IC_50_ MTgal	IC_50_ATPglu/IC_50_ ATPgal	IC_50_MTglu/IC_50_ATPglu
	glu	gal	glu	gal			
Imatinib	>100	>100	19.5	22.3	n.d.	0.87	>5.1
Sorafenib	30.7	19.4	17.1	5.40	1.6	3.2	1.8

Abbreviations: glu, glucose; gal, galactose; MT, membrane toxicity; IC_50_, concentration of toxicant with 50% of maximal effect.

**Table 2:** Primer sequences used for quantitative real-time PCR amplification.

Target Gene	Forward Primer 5′--------->3′ Reverse Primer 5′--------->3′	Species
*Sod1*	AGATGACTTGGGCAAAGGTG CAATCCCAATCACACCACAA	rat
*Sod2*	CTGGACAAACCTGAGCCCTA GAACCTTGGACTCCCACAGA	rat
*18s*	TTGCTGACAGGATGCAGAAG CAGTGAGGCCAGGATAGAGC	rat
*ND1*	GCAGCTTAACATTCCGCCCAATCA TACTGGTTGGCCTCCGATTCATGT	rat
*36B4*	TGTGGGTGATCTGGTGATTGTGGT AGGCATTTCAGGATACGCTCAGCA	rat

## Data Availability

The data presented in this study are available from the corresponding author on a reasonable request.

## References

[1] DeVita V.T., Chu E. (2008). A history of cancer chemotherapy. Cancer Res..

[2] Kantarjian H., Sawyers C., Hochhaus A., Guilhot F., Schiffer C., Gambacorti-Passerini C., Niederwieser D., Resta D., Capdeville R., Zoellner U. (2002). Hematologic and cytogenetic responses to imatinib mesylate in chronic myelogenous leukemia. N. Engl. J. Med..

[3] Goldman J.M., Melo J.V. (2001). Targeting the BCR-ABL tyrosine kinase in chronic myeloid leukemia. N. Engl. J. Med..

[4] Varga Z.V., Ferdinandy P., Liaudet L., Pacher P. (2015). Drug-induced mitochondrial dysfunction and cardiotoxicity. Am. J. Physiol. Heart Circ. Physiol..

[5] Zhu Y.J., Zheng B., Wang H.Y., Chen L. (2017). New knowledge of the mechanisms of sorafenib resistance in liver cancer. Acta Pharmacol. Sin..

[6] Hartmann J.T., Haap M., Kopp H.G., Lipp H.P. (2009). Tyrosine kinase inhibitors—A review on pharmacology, metabolism and side effects. Curr. Drug Metab..

[7] Force T., Krause D.S., Van Etten R.A. (2007). Molecular mechanisms of cardiotoxicity of tyrosine kinase inhibition. Nat. Rev. Cancer.

[8] Kerkela R., Grazette L., Yacobi R., Iliescu C., Patten R., Beahm C., Walters B., Shevtsov S., Pesant S., Clubb F.J. (2006). Cardiotoxicity of the cancer therapeutic agent imatinib mesylate. Nat. Med..

[9] Escalante C.P., Chang Y.C., Liao K., Rouleau T., Halm J., Bossi P., Bhadriraju S., Brito-Dellan N., Sahai S., Yusuf S.W. (2016). Meta-analysis of cardiovascular toxicity risks in cancer patients on selected targeted agents. Support. Care Cancer Off. J. Multinatl. Assoc. Support. Care Cancer.

[10] Chen M.H., Kerkela R., Force T. (2008). Mechanisms of cardiac dysfunction associated with tyrosine kinase inhibitor cancer therapeutics. Circulation.

[11] Chu T.F., Rupnick M.A., Kerkela R., Dallabrida S.M., Zurakowski D., Nguyen L., Woulfe K., Pravda E., Cassiola F., Desai J.L. (2007). Cardiotoxicity associated with tyrosine kinase inhibitor sunitinib. Lancet.

[12] Mellor H.R., Bell A.R., Valentin J.P., Roberts R.R. (2011). Cardiotoxicity associated with targeting kinase pathways in cancer. Toxicol. Sci..

[13] Orphanos G.S., Ioannidis G.N., Ardavanis A.G. (2009). Cardiotoxicity induced by tyrosine kinase inhibitors. Acta Oncol..

[14] Schmidinger M., Zielinski C.C., Vogl U.M., Bojic A., Bojic M., Schukro C., Ruhsam M., Hejna M., Schmidinger H. (2008). Cardiac toxicity of sunitinib and sorafenib in patients with metastatic renal cell carcinoma. J. Clin. Oncol. Off. J. Am. Soc. Clin. Oncol..

[15] Bouitbir J., Panajatovic M.V., Frechard T., Roos N.J., Krähenbühl S. (2020). Imatinib and Dasatinib Provoke Mitochondrial Dysfunction Leading to Oxidative Stress in C2C12 Myotubes and Human RD Cells. Front. Pharmacol..

[16] Damaraju V.L., Kuzma M., Cass C.E., Putman C.T., Sawyer M.B. (2018). Multitargeted kinase inhibitors imatinib, sorafenib and sunitinib perturb energy metabolism and cause cytotoxicity to cultured C2C12 skeletal muscle derived myotubes. Biochem. Pharmacol..

[17] Mingard C., Paech F., Bouitbir J., Krähenbühl S. (2018). Mechanisms of toxicity associated with six tyrosine kinase inhibitors in human hepatocyte cell lines. J. Appl. Toxicol. JAT.

[18] Paech F., Bouitbir J., Krähenbühl S. (2017). Hepatocellular Toxicity Associated with Tyrosine Kinase Inhibitors: Mitochondrial Damage and Inhibition of Glycolysis. Front. Parmacology.

[19] Paech F., Mingard C., Grünig D., Abegg V.F., Bouitbir J., Krähenbühl S. (2018). Mechanisms of mitochondrial toxicity of the kinase inhibitors ponatinib, regorafenib and sorafenib in human hepatic HepG2 cells. Toxicology.

[20] Yan H., Du J., Chen X., Yang B., He Q., Yang X., Luo P. (2019). ROS-dependent DNA damage contributes to crizotinib-induced hepatotoxicity via the apoptotic pathway. Toxicol. Appl. Pharmacol..

[21] Will Y., Dykens J.A., Nadanaciva S., Hirakawa B., Jamieson J., Marroquin L.D., Hynes J., Patyna S., Jessen B.A. (2008). Effect of the multitargeted tyrosine kinase inhibitors imatinib, dasatinib, sunitinib, and sorafenib on mitochondrial function in isolated rat heart mitochondria and H9c2 cells. Toxicol. Sci..

[22] Bouitbir J., Alshaikhali A., Panajatovic M.V., Abegg V.F., Paech F., Krähenbühl S. (2019). Mitochondrial oxidative stress plays a critical role in the cardiotoxicity of sunitinib: Running title: Sunitinib and oxidative stress in hearts. Toxicology.

[23] French K.J., Coatney R.W., Renninger J.P., Hu C.X., Gales T.L., Zhao S., Storck L.M., Davis C.B., McSurdy-Freed J., Chen E. (2010). Differences in effects on myocardium and mitochondria by angiogenic inhibitors suggest separate mechanisms of cardiotoxicity. Toxicol. Pathol..

[24] Marroquin L.D., Hynes J., Dykens J.A., Jamieson J.D., Will Y. (2007). Circumventing the Crabtree effect: Replacing media glucose with galactose increases susceptibility of HepG2 cells to mitochondrial toxicants. Toxicol. Sci..

[25] Kamalian L., Chadwick A.E., Bayliss M., French N.S., Monshouwer M., Snoeys J., Park B.K. (2015). The utility of HepG2 cells to identify direct mitochondrial dysfunction in the absence of cell death. Toxicol. Int. J. Publ. Assoc. BIBRA.

[26] Swiss R., Niles A., Cali J.J., Nadanaciva S., Will Y. (2013). Validation of a HTS-amenable assay to detect drug-induced mitochondrial toxicity in the absence and presence of cell death. Toxicol. Int. J. Publ. Assoc. BIBRA.

[27] Haegler P., Joerin L., Krahenbuhl S., Bouitbir J. (2017). Hepatocellular Toxicity of Imidazole and Triazole Antimycotic Agents. Toxicol. Sci..

[28] Drose S., Brandt U. (2012). Molecular mechanisms of superoxide production by the mitochondrial respiratory chain. Adv. Exp. Med. Biol..

[29] Felser A., Blum K., Lindinger P.W., Bouitbir J., Krahenbuhl S. (2013). Mechanisms of hepatocellular toxicity associated with dronedarone—A comparison to amiodarone. Toxicol. Sci..

[30] Fernandez-Checa J.C., Kaplowitz N. (2005). Hepatic mitochondrial glutathione: Transport and role in disease and toxicity. Toxicol. Appl. Pharmacol..

[31] Schafer F.Q., Buettner G.R. (2001). Redox environment of the cell as viewed through the redox state of the glutathione disulfide/glutathione couple. Free Radic. Biol. Med..

[32] Orlicka-Plocka M., Gurda-Wozna D., Fedoruk-Wyszomirska A., Wyszko E. (2020). Circumventing the Crabtree effect: Forcing oxidative phosphorylation (OXPHOS) via galactose medium increases sensitivity of HepG2 cells to the purine derivative kinetin riboside. Apoptosis.

[33] Birch M., Morgan P.E., Handley S., Ho A., Ireland R., Flanagan R.J. (2013). Simple methodology for the therapeutic drug monitoring of the tyrosine kinase inhibitors dasatinib and imatinib. Biomed. Chromatogr. BMC.

[34] Ma W., Liu M., Liang F., Zhao L., Gao C., Jiang X., Zhang X., Zhan H., Hu H., Zhao Z. (2020). Cardiotoxicity of sorafenib is mediated through elevation of ROS level and CaMKII activity and dysregulation of calcium homoeostasis. Basic Clin. Pharmacol. Toxicol..

[35] Kawabata M., Umemoto N., Shimada Y., Nishimura Y., Zhang B., Kuroyanagi J., Miyabe M., Tanaka T. (2015). Downregulation of stanniocalcin 1 is responsible for sorafenib-induced cardiotoxicity. Toxicol. Sci..

[36] Jensen B.C., Parry T.L., Huang W., Ilaiwy A., Bain J.R., Muehlbauer M.J., O’Neal S.K., Patterson C., Johnson G.L., Willis M.S. (2017). Non-Targeted Metabolomics Analysis of the Effects of Tyrosine Kinase Inhibitors Sunitinib and Erlotinib on Heart, Muscle, Liver and Serum Metabolism In Vivo. Metabolites.

[37] Stuhlmiller T.J., Zawistowski J.S., Chen X., Sciaky N., Angus S.P., Hicks S.T., Parry T.L., Huang W., Beak J.Y., Willis M.S. (2017). Kinome and Transcriptome Profiling Reveal Broad and Distinct Activities of Erlotinib, Sunitinib, and Sorafenib in the Mouse Heart and Suggest Cardiotoxicity from Combined Signal Transducer and Activator of Transcription and Epidermal Growth Factor Receptor Inhibition. J. Am. Heart Assoc..

[38] Jacob F., Yonis A.Y., Cuello F., Luther P., Schulze T., Eder A., Streichert T., Mannhardt I., Hirt M.N., Schaaf S. (2016). Analysis of Tyrosine Kinase Inhibitor-Mediated Decline in Contractile Force in Rat Engineered Heart Tissue. PloS ONE.

[39] Haas N.B., Manola J., Ky B., Flaherty K.T., Uzzo R.G., Kane C.J., Jewett M., Wood L., Wood C.G., Atkins M.B. (2015). Effects of Adjuvant Sorafenib and Sunitinib on Cardiac Function in Renal Cell Carcinoma Patients without Overt Metastases: Results from ASSURE, ECOG 2805. Clin. Cancer Res. Off. J. Am. Assoc. Cancer Res..

[40] Zhang C., Liu Z., Bunker E., Ramirez A., Lee S., Peng Y., Tan A.C., Eckhardt S.G., Chapnick D.A., Liu X. (2017). Sorafenib targets the mitochondrial electron transport chain complexes and ATP synthase to activate the PINK1-Parkin pathway and modulate cellular drug response. J. Biol. Chem..

[41] Djafarzadeh S., Jakob S.M. (2017). High-resolution Respirometry to Assess Mitochondrial Function in Permeabilized and Intact Cells. J. Vis. Exp..

[42] Long Q., Huang L., Huang K., Yang Q. (2019). Assessing Mitochondrial Bioenergetics in Isolated Mitochondria from Mouse Heart Tissues Using Oroboros 2k-Oxygraph. Methods Mol. Biol..

[43] Herbrink M., de Vries N., Rosing H., Huitema A.D., Nuijen B., Schellens J.H., Beijnen J.H. (2016). Quantification of 11 Therapeutic Kinase Inhibitors in Human Plasma for Therapeutic Drug Monitoring Using Liquid Chromatography Coupled with Tandem Mass Spectrometry. Ther. Drug Monit..

[44] Huynh H.H., Pressiat C., Sauvageon H., Madelaine I., Maslanka P., Lebbe C., Thieblemont C., Goldwirt L., Mourah S. (2017). Development and Validation of a Simultaneous Quantification Method of 14 Tyrosine Kinase Inhibitors in Human Plasma Using LC-MS/MS. Ther. Drug Monit..

[45] Pesta D., Gnaiger E. (2012). High-resolution respirometry: OXPHOS protocols for human cells and permeabilized fibers from small biopsies of human muscle. Methods Mol. Biol..

[46] Kuznetsov A.V., Veksler V., Gellerich F.N., Saks V., Margreiter R., Kunz W.S. (2008). Analysis of mitochondrial function in situ in permeabilized muscle fibers, tissues and cells. Nat. Protoc..

[47] Gnaiger E., Kuznetsov A.V. (2002). Mitochondrial respiration at low levels of oxygen and cytochrome c. Biochem. Soc. Trans..

[48] Rao X., Huang X., Zhou Z., Lin X. (2013). An improvement of the 2^(-delta delta CT) method for quantitative real-time polymerase chain reaction data analysis. Biostat. Bioinform. Biomath..

[49] Quiros P.M., Goyal A., Jha P., Auwerx J. (2017). Analysis of mtDNA/nDNA Ratio in Mice. Curr. Protoc. Mouse Biol..

[50] Lam J., Katti P., Biete M., Mungai M., AshShareef S., Neikirk K., Garza Lopez E., Vue Z., Christensen T.A., Beasley H.K. (2021). A Universal Approach to Analyzing Transmission Electron Microscopy with ImageJ. Cells.

